# From survival to positive childbirth experiences: An integrative review of facility-based maternity care in South-East Asia

**DOI:** 10.18332/ejm/240344

**Published:** 2026-10-22

**Authors:** Evanjalin Dhamirah Arulpragassam, Meetha Kalcik, Preet Phull, Malin Bogren, Kerstin Erlandsson, Lise-Lotte Franklin, Catrin Borneskog

**Affiliations:** 1 School of Health and Welfare, Dalarna University, Falun, Sweden; 2 Institute of Health and Care Sciences, University of Gothenburg, Gothenburg, Sweden

**Keywords:** childbirth experience, maternal and newborn care, quality of care, South-East Asia, integrative review

## Abstract

**INTRODUCTION:**

Women's childbirth experiences provide information for improving the quality of maternity care. This study explored opportunities and challenges related to achieving a positive childbirth experience among women giving birth in health facilities in the WHO-defined South-East Asia Region.

**METHODS:**

An integrative review was conducted following the five stages described by Whittemore and Knafl. PubMed and CINAHL were searched, supplemented by reference list screening, for studies published in English between 2012 and 2023. Studies from the WHO-defined South-East Asia Region focusing on women's experiences of facility-based childbirth were included. Methodological quality was assessed using Pluye's Mixed-Methods Appraisal Tool, and data were analyzed using inductive content analysis.

**RESULTS:**

A total of 2886 records were identified. After duplicate removal and screening, 104 full-text articles were assessed for eligibility and 26 studies were included. The overarching theme was: ‘Women prioritize survival over their childbirth experiences when giving birth in health facilities’. Three generic categories were identified: Quality of care, Emotional and physical support, and Adherence to women's rights principles. Positive experiences were associated with competent care providers, supportive companionship, effective communication, and respectful care. Challenges included poor communication, limited emotional support, inadequate resources, and experiences of mistreatment and discrimination.

**CONCLUSIONS:**

Women prioritized the survival of themselves and their newborns above their childbirth experiences. Positive childbirth experiences may be supported by high-quality care, effective communication, companionship during labor and birth, and respectful maternity care. Future research should explore strategies to strengthen respectful maternity care, emotional support, and evidence-based practices across maternity care settings.

## INTRODUCTION

Maternal health and reducing maternal and newborn mortality and morbidity are essential components of global health goals, as reflected in the Sustainable Development Goals (SDGs)^1^. While expanding access to maternal healthcare services remains a critical objective, recent evidence suggests that the quality of care within health facilities is a more significant determinant of maternal and neonatal outcomes than mere access^2,3^. One alarming consequence of poor-quality care is that women may be reluctant to utilize healthcare facilities, particularly for childbirth, in subsequent pregnancies^4^. Despite access to healthcare, persistent barriers to obtaining safe, effective, timely, and women-centered care lead women to consider alternative options^5^. This has profound implications for maternal and neonatal health outcomes, as women’s decisions regarding where to give birth are shaped by their previous experiences and perceptions of the care they received^6,7^. In the South-East Asian region, maternal and newborn healthcare is provided by a range of healthcare providers, including midwives, doctors, nurses, nurse-midwives, and auxiliary nurse midwives. The maternal mortality ratio in the South-East Asian region in 2017 was 152 per 100000 live births, while the neonatal mortality rate in 2019 was 20 per 1000 live births^8^. The proportion of births attended by skilled healthcare providers ranges from 57% to 100% across the region, with Thailand, Democratic People’s Republic of Korea, Sri Lanka, and the Maldives reporting that more than 99% of births are attended by skilled healthcare providers^9^. The WHO-defined South-East Asia Region was selected because it includes countries with diverse health systems, levels of maternal and newborn health outcomes, and models of maternity care provision. Despite substantial improvements in maternal and newborn survival across the region, important inequalities remain regarding access to and quality of facility-based maternity care. Understanding women’s childbirth experiences across this region may therefore provide valuable insights into opportunities and challenges for achieving positive childbirth experiences^10^.

According to the World Health Organization (WHO), a positive childbirth experience is defined as ‘one that fulfils or exceeds a woman’s prior personal and sociocultural beliefs and expectations, including giving birth to a healthy newborn in a clinically and psychologically safe environment with continuity of practical and emotional support from a birth companion(s) and kind, technically competent clinical staff^10^. Hence, understanding women’s childbirth experiences can contribute to improving the quality of care by identifying actions needed to strengthen healthcare services and highlighting areas requiring governmental investment^11^. With this background, the aim of this study was to explore the opportunities and challenges associated with achieving a positive childbirth experience for women giving birth in health facilities. By addressing the identified challenges and building on the identified opportunities, it is possible to work towards a healthcare system that ensures every woman’s childbirth experience is safe, empowering, and positive^5^, thereby contributing to the overall well-being of women and their families.

## METHODS

### Design

The study is an integrative literature review according to the method described by Whittemore and Knafl^12^. Integrative literature review incorporates both quantitative and qualitative data and mixed-methods studies. Problem identification, literature search, data evaluation, data analysis and presentation of results are the five stages in this method^12^.

### Literature search

Eligible studies included qualitative, quantitative and mixed-methods studies reporting women’s experiences of facility-based childbirth and maternity care. The inclusion and exclusion criteria were developed using the PEOS (Population, Exposure^13^, Outcome, Study type) framework (Table 1). Studies were eligible if they were published in English between 2012 and 2023 and reported women’s experiences, perceptions, satisfaction, support, respectful maternity care, communication, or quality of care related to facility-based childbirth. Studies conducted in countries belonging to the WHO-defined South-East Asia Region were eligible for inclusion. Review articles, editorials, commentaries, conference abstracts, study protocols, and studies that did not report women’s childbirth experiences, were excluded. The search strategy was developed in accordance with the Preferred Reporting Items for Systematic Reviews and Meta-Analyses (PRISMA) guidelines^14^ and with the assistance of a librarian at Dalarna University. PubMed and CINAHL were searched, in addition to reference list screening. The search strategy combined controlled vocabulary terms and free-text keywords related to intrapartum care (e.g. support and clinical management during labor and childbirth) and/or postpartum care (e.g. support for women and newborns, neonatal examinations, breastfeeding, and postpartum contraception). Search terms were adapted to the indexing systems and search functionalities of each database. Boolean operators (AND/OR) were used to combine search concepts. The full search strategy for each database is presented in the Supplementary file. The first author conducted the title and abstract screening, full-text eligibility assessment, and data extraction. Any uncertainties regarding study eligibility or interpretation were discussed with the co-authors until consensus was reached. A total of 2864 and 22 additional records were identified from CINAHL, PubMed, and reference list screening. The total records identified was then 2886. The additional sources consisted of reference lists from review articles identified through the database searches. First, 39 duplicate records were removed. The remaining records were then screened based on their titles and abstracts. Subsequently, 104 full-text articles were assessed for eligibility, of which 78 were excluded. This process resulted in 26 included articles in the review (Figure 1). The excluded articles and the reasons for exclusion are presented in the Supplementary file.

**Table 1:** Inclusion and exclusion criteria used for study selection in this integrative review of women’s experiences of facility-based maternity care in South-East Asia. Eligibility criteria were based on the PEOS framework and applied to peer-reviewed original research articles published in English between 2012 and 2023

*Eligibility criteria*	*Inclusion criteria*	*Exclusion criteria*
**Participants**	Women with experience of facility-based childbirth. Studies conducted in the WHO South-East Asia Region	Immigrants Care providers
**Exposure**	Midwifery or maternity care provided by skilled birth attendants as defined by Renfrew et al.13. Public and private health facilities	Traditional birth attendant Home birth/unknown place of childbirth Special circumstances (COVID, natural disasters)
**Outcome**	Childbirth experiences, postnatal experiences, women’s perceptions and satisfaction with care, respectful maternity care, communication and support during childbirth	Antenatal experience Abortion experience
**Study type**	Qualitative, quantitative and mixed-methods studies; peer-reviewed original articles; published in English between 2012 and 2023	Review articles Languages other than English Published outside 2012–2023

**Figure 1:**
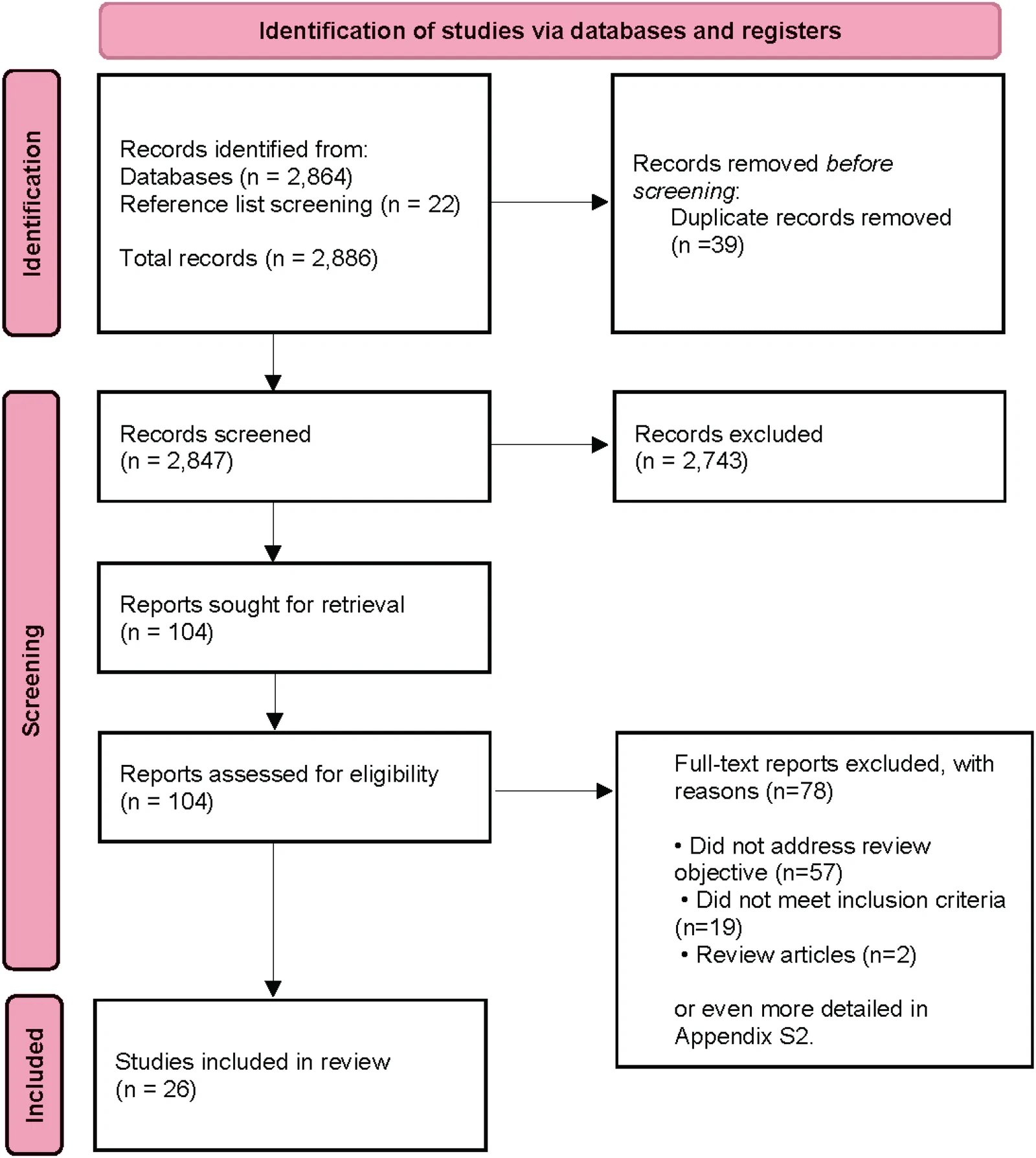
Prisma flow chart of selection of studies

### Data evaluation

Pluye’s Mixed Methods Appraisal Tool (MMAT) was used to assess the quality of the selected articles. The MMAT is a critical appraisal tool comprising 25 criteria across five study categories: qualitative studies, quantitative randomized controlled trials, quantitative non-randomized studies, quantitative descriptive studies, and mixed-methods studies^15^. The quality assessment was conducted independently by the first two authors. Methodological quality was assessed using the Mixed Methods Appraisal Tool (MMAT). The appraisal focused on methodological rigor, transparency of reporting, appropriateness of the study design, data collection and analysis procedures, and potential sources of bias. For descriptive purposes, studies were categorized as having high (76–100%), moderate (51–75%), or low (≤50%) methodological quality. The appraisal was used to identify methodological strengths and limitations of the included studies and to support interpretation of the findings rather than to exclude studies based on a predefined cutoff value (Table 3). Following the quality assessment, all 26 articles met the inclusion threshold and were retained for analysis.

### Data analysis

An inductive content analysis according to Elo and Kyngäs^16^ was performed manually. The included articles were read several times, and text units reflecting the opportunities and challenges associated with a positive childbirth experience for women giving birth in health facilities were extracted and transferred to an Excel spreadsheet. The extracted text was then read repeatedly, and relevant text units were assigned codes. The codes were compared based on their similarities and differences and grouped into subcategories. To capture both positive and challenging childbirth experiences, opportunities and challenges relating to the same phenomenon were organized within the same category. The coded text units were condensed and further analyzed to identify patterns and relationships. Subcategories were subsequently grouped into generic categories, which were then abstracted into a main category. An example of the qualitative content analysis process used in the integrative review of the 26 studies^17-42^ is presented in Table 2.

**Table 2:** Example of the qualitative content analysis process used in the integrative review of 26 studies on facility-based maternity care and childbirth experiences in South-East Asia. The examples demonstrate how text extracted from a primary study was condensed into a code, subcategory, and generic category during data analysis

*Article references*	*Text*	*Code*	*Subcategory*	*Generic category*
7	Lack of privacy was attributed to the absence of curtains separating women in the labor room^18^ and the high number of healthcare providers present during physical examinations^41^.	Lack of privacy during childbirth	Respectful maternity care	Adherence to principles of women’s rights
24, 41	Women also encountered language barriers that hindered communication.	Communication barriers	Communication	Emotional and physical support
18	*‘We did not have any bed, in the corridor itself. So when I was very tired, my mother-in-law spread a shawl on the floor for me to rest for some time … I wish they had given us a bed.’*	Insufficient bed availability	Environment and resource availability	Quality of care
20	*‘When it is very painful, I bit his clothes. I feel so calm when he stands beside me. In every delivery process, he is just enough. He massages and prays for me continuously. And it makes me strong.’*	Emotional support from birth companion	Birth companion	Emotional and physical support

**Table 3:** Characteristics of the 26 studies included in the integrative review of facility-based maternity care and childbirth experiences in South-East Asia (2012–2023), including author, publication year, country, study design, study aim, and methodological appraisal outcomes

*Author* *Year* *Title*	*Country, Design*	*Objective*	Methodological appraisal outcomes*
Maung et al.^17^ 2020 A qualitative study on acceptability of the mistreatment of women during childbirth in Myanmar	Myanmar Qualitative	To explore the acceptability of mistreatment during childbirth in Myanmar according to women and healthcare providers, and to understand how gender power relations influence mistreatment during childbirth.	High quality
Nair et al.^25^ 2019 An Exploratory Study to Assess the Factors Contributing to Maternal Childbirth Satisfaction with Delivery Services among Postnatal Mothers at Selected Hospital in Mohali, Punjab	India Quantitative	To identify the factors contributing to maternal childbirth satisfaction with delivery services among postnatal mothers.	High quality
Rahman et al.^26^ 2018 Are the urban poor satisfied with health care services for child delivery? Evidence from an Urban Primary Health Care (UPHC) Project in Bangladesh	Bangladesh Quantitative	To assess the level of satisfaction with delivery care and its associated factors among the mothers who delivered their child at health facilities in the urban areas of Bangladesh.	High quality
Shrestha et al.^27^ 2021 Assessment of Client Satisfaction in Labour and Delivery Services in a Tertiary Hospital in Western Nepal	Nepal Quantitative	To assess client satisfaction with maternity care in a tertiary hospital in the western region of Nepal.	High quality
Rajkumari et al.^28^ 2021 Assessment of respectful maternity care during childbirth: Experiences among mothers in Manipur	India Quantitative	To determine the prevalence and types of disrespect and abuse among mothers during childbirth and its associated factors.	High quality
Raj et al.^29^ 2017 Associations Between Mistreatment by a Provider during Childbirth and Maternal Health Complications in Uttar Pradesh, India	India Quantitative	To assess associations between mistreatment by a provider during childbirth and maternal complications in Uttar Pradesh, India.	High quality
Jha et al.^18^ 2016 Cashless childbirth, but at a cost: A grounded theory study on quality of intrapartum care in public health facilities in India	India Qualitative	To explore and understand the perceptions and experiences of women regarding quality of care received during childbirth in public health facilities.	High quality
Wickramasinghe et al.^30^ 2019 Client perceived quality of the postnatal care provided by public sector specialized care institutions following a normal vaginal delivery in Sri Lanka: a cross-sectional study	Sri Lanka Quantitative	To assess the quality of the regular postnatal care provided by the public sector specialized institutions in the district of Colombo, Sri Lanka, following a normal vaginal delivery, from the mothers’perspective, and the factors that are associated with their perceptions.	High quality
Rishard et al.^31^ 2021 Correlation among experience of person-centered maternity care, provision of care and women’s satisfaction: Cross sectional study in Colombo, Sri Lanka	Sri Lanka Quantitative	To explore different domains of quality of care–namely degree of implementation of PCMC (person-centered maternity care), key indicators of provision of care, and women’s satisfaction with maternal care–in a tertiary care center in Sri Lanka, and to analyze correlations among these three domains, as well as key factors associated with each domain.	High quality
Nawab et al.^32^ 2019 Disrespect and abuse during facility-based childbirth and its sociodemographic determinants-A barrier to healthcare utilization in rural population	India Quantitative	To assess prevalence and sociodemographic determinants of DA (disrespect abuse) experienced during facility-based childbirth.	High quality
Brunell et al.^33^ 2022 Effect of a perinatal care quality improvement package on patient satisfaction: a secondary outcome analysis of a cluster-randomized controlled trial	Nepal Quantitative	To investigate the effect of a quality improvement (QI) package on patient satisfaction of perinatal care.	Moderate quality
Erfina et al.^19^ 2019 Exploring Indonesian adolescent women’s healthcare needs as they transition to motherhood: A qualitative study	Indonesia Qualitative	To explore adolescent mothers’ postnatal inpatient experiences and healthcare needs as they moved towards their maternal roles.	High quality
Mehata et al.^34^ 2017 Factors determining satisfaction among facility-based maternity clients in Nepal	Nepal Quantitative	To identify the determinants of client satisfaction with maternity care in Nepal using data from a nationally representative health facility survey.	High quality
Astuti et al.^35^ 2019 Intrapartum care satisfaction at three levels of healthcare facilities in Jakarta	Indonesia Quantitative	To identify maternal satisfaction with childbirth delivery services at three levels of healthcare facilities in Jakarta, Indonesia.	High quality
Rachmawati^20^ 2012 Maternal reflection on labour pain management and influencing factors	Indonesia Qualitative	To describe the mothers’ reflection on the labor pain management experienced in the Indonesian setting, and any influencing factors on their perception of pain and care provided.	High quality
Gurung et al.^36^ 2022 Mistreatment during childbirth and postnatal period reported by women in Nepal -a multicentric prevalence study	Nepal Quantitative	To measure the magnitude of mistreatment of women during childbirth in health institutions in Nepal.	High quality
Doria et al.^21^ 2019 Mothers’ experiences of quality of care and potential benefits of implementing the WHO safe childbirth checklist: a case study of Aceh Indonesia	Indonesia Qualitative	To translate the perception of quality of care from Acehnese women who gave institutional birth in this province.	High quality
Sapkota et al.^22^ 2014 Nepalese mothers’ experiences of care in labor	Nepal Qualitative	To explore mothers’ experiences of the care received while giving birth in hospital settings in Nepal.	High quality
Montagu et al.^37^ 2019 Patient-experience during delivery in public health facilities in Uttar Pradesh, India	India Quantitative	To understand the variation of women’s experiences in different levels of government facilities.	High quality
Iyer et al.^23^ 2020 Perceptions of quality of care during birth at private Chiranjeevi facilities in Gujarat: lessons for Universal Health Coverage	India Qualitative	To qualitatively explore the perception of quality of care during childbirth among mothers who had recently given birth in CY partnering private facilities.	High quality
Gurung et al.^38^ 2021 Respectful maternal and newborn care: measurement in one EN-BIRTH study hospital in Nepal	Nepal Quantitative	To analyze exit survey-reported experience of care for women after hospital birth. To describe women’s exit survey-reported coverage of facility based newborn care practices around the time of birth. To conduct multivariate regression analysis regarding determinants of reporting by women, including mode of birth, and demographic and social characteristics.	High quality
Jha et al.^39^ 2017 Satisfaction with childbirth services provided in public health facilities: results from a cross- sectional survey among postnatal women in Chhattisgarh, India	India Quantitative	To measure postnatal Indian women’s satisfaction with childbirth services at selected public health facilities in Chhattisgarh, India.	High quality
Diamond-Smith et al.^40^ 2016 The relationship between women’s experiences of mistreatment at facilities during childbirth, types of support received and person providing the support in Lucknow, India	India Quantitative	To explore women’s reported experiences of mistreatment during childbirth quantitatively, and how these varied by specific types of support available and provided by specific individuals.	High quality
Perera et al.^24^ 2018 ‘When helpers hurt’: women’s and midwives’ stories of obstetric violence in state health institutions, Colombo district, Sri Lanka	Sri Lanka Qualitative	To break the traditional culture of silence around obstetric violence and bring attention to the resulting implications for quality of care and patient trust in obstetric care facilities or providers.	High quality
Ghimire et al.^41^ 2021 Women’s Experience of Disrespect and Abuse during Institutional Delivery in Biratnagar, Nepal	Nepal Quantitative	To find out the women’s experience of disrespect and abuse during institutional delivery.	High quality
Deki and Wangmo^42^ 2020 Women’s Views and Experience of Respectful Maternity Care While Delivering in Three Regional Referral Hospitals of Bhutan	Bhutan Quantitative	To explore the attitudes, views, behaviors and emotional experiences of women related to labor and childbirth and to describe women’s satisfaction with respectful maternity care in three regional hospitals of Bhutan.	High quality

* Methodological quality: High (76–100%); Moderate (51–75%); Low (≤50%).

## RESULTS

Twenty-six articles were included in the review. The findings were derived from both qualitative (n=8) and quantitative (n=18) studies. Qualitative studies primarily contributed women’s descriptions and experiences of childbirth, whereas quantitative studies provided information on the prevalence and distribution of these experiences. The studies were conducted in Bangladesh (n=1), Bhutan (n=1), India (n=9), Indonesia (n=4), Myanmar (n=1), Nepal (n=7), and Sri Lanka (n=3). No articles from Democratic People’s Republic of Korea, the Maldives, Thailand, or Timor-Leste met the inclusion criteria. Of the 26 included articles, 8 employed a qualitative design^17-24^, while 18 employed a quantitative design^25-42^. The characteristics of the included studies are presented in Table 3. The analysis generated one main category: ‘Women prioritize survival over their childbirth experiences when giving birth in health facilities’. This main category emerged across studies from multiple countries in the WHO-defined South-East Asia Region and was reflected in both qualitative and quantitative findings. Women consistently described survival of themselves and their newborns as the most important outcome of childbirth, even when they experienced poor communication, inadequate support, limited privacy, or disrespectful treatment. Across studies, women frequently expressed gratitude for achieving a safe birth despite challenges encountered during labor and childbirth. The main category comprised three generic categories: 1) Quality of care, 2) Emotional and physical support, and 3) Adherence to women’s rights principles. An overview of the main category, generic categories, and subcategories is presented in Table 4. The articles contributing to each category are presented in Table 5.

**Table 4:** Analytical framework derived from the integrative review of 26 studies on women’s childbirth experiences in health facilities across South-East Asia (2012–2023), illustrating the main category, generic categories, and subcategories identified through qualitative content analysis

**Main category**	Women prioritize survival over their childbirth experiences when giving birth in health facilities		
**Generic categories**	Quality of care	Emotional and physical support	Adherence to principles of women’s rights
**Subcategories**	Organization of care Care practices Environment and resource availability	Strengthening women’s capabilities Birth companion Communication	Respectful maternity care Equality of care

**Table 5:** Distribution of the 26 included studies across the identified categories and subcategories of childbirth experiences in facility-based maternity care in South-East Asia (2012–2023), including represented countries

*Main category*	*Generic categories*	*Subcategories and the articles included*	*Countries represented*
Women prioritize survival over experiences when giving birth in health facilities	Quality of care	Organization of care^17,18,21-28,31,32,34,35,39,41,42^	Myanmar India Bangladesh Nepal Sri Lanka Indonesia
		Care practices^18-23,27,29-31,33,34,36-39,41^	India Indonesia Nepal Sri Lanka
		Environment and resource availability^18,21-23,28,30,34^	India Indonesia Nepal Sri Lanka
	Emotional and physical support	Strengthening woman’s capabilities^17-23,26,28,29,31,35,38,40^	Bangladesh India Indonesia Myanmar Nepal Sri Lanka
		Birth companion^18,20-22,28,31,33,41^	India Indonesia Nepal
		Communication^18-22,24,26,27,29-31,33,34,36,38,40-42^	Bangladesh Bhutan India Indonesia Nepal Sri Lanka
	Adherence to principles of women’s rights	Respectful maternity care^17,18,21-24,27-33,36,38,39,41,42^	Bhutan India Indonesia Myanmar Nepal Sri Lanka
		Equality of care^17,18,23,24,29,32,36,38,41,42^	Bhutan India Myanmar Nepal Sri Lanka

### Quality of care

Opportunities and challenges related to the provision of quality care encompassed various aspects of healthcare and are described under the following subcategories: 1) Organization of care, 2) Care practices, and 3) Environment and resource availability.

### Organization of care

Seventeen studies contributed to this subcategory^17,18,21-28,31,32,34,35,39,41,42^. An opportunity for a positive childbirth experience based on the organization of care was related to the availability, accessibility, and affordability of services provided by healthcare providers in health facilities^17,18,21-28,31,32,34,35,39,41,42^. In four studies conducted in Bangladesh, India, Sri Lanka, and Nepal^26,31,32,39^, between 68.7% and 86.9% of women (sample sizes ranging from 305 to 3949 participants) reported satisfaction with the overall childbirth services. One study from India^25^ reported that 34.4% of 180 women were satisfied with the services, while the remaining women reported neutral perceptions; none expressed dissatisfaction. Some studies indicated that women were satisfied with the competency of healthcare providers^18,23,26^, their availability throughout childbirth^22^, and the time spent with them^27^.

Other studies reported that women experienced adverse events during childbirth^24,42^. A challenge to achieving a positive childbirth experience was the inattentiveness of healthcare providers during labour^21,24,41^. Women believed that free maternity services^23^, the continuous availability of healthcare providers throughout labour^21,28,42^, and following healthcare providers’ instructions^17^ would contribute to a positive childbirth experience. Women who did not have to pay for services reported higher levels of satisfaction^34^. Despite negative experiences with the services or healthcare providers, women expressed gratitude toward the health system and the providers for helping them achieve a safe birth and survive the childbirth process^23,24^:

*‘Our [my] delivery was nice, child is nice, lady is saved; it means everything was complete... (there was) no other problem, that’s how we [mothers] think.* ^23^

### Care practices

Seventeen studies contributed to this subcategory^18-23,27,29-31,^ ^33,34,36-39,41^. Women valued practices that contributed to a safe birth outcome and often accepted interventions they believed were necessary to protect their own lives or the lives of their newborns^18-23,27,29-31,33,34,36-39,41^. Opportunities and challenges in care practices included health education, medical procedures, and interventions related to maternal and newborn care. Opportunities for a positive childbirth experience were identified when care was provided in accordance with evidence-based practices^18,39^, national guidelines^20,30^, and good clinical practices, such as avoiding unnecessary caesarean sections^22^. In Nepal, where a perinatal care quality improvement package was implemented, the percentage of women reporting satisfaction with care increased from 58% to 62% (n=54919)^33^. Health education varied and covered topics such as breastfeeding, newborn care, self-care, family planning, and follow-up care for women and children^19,21,22,33,34,36-38^. Challenges to a positive childbirth experience were related to newborn care, pain management, and harmful practices. Challenges in newborn care included inadequate attention to newborn needs, such as a lack of skin-to-skin contact or kangaroo mother care^30,38^, the absence of neonatal examinations conducted in the mother’s presence^36^, inadequate breastfeeding support^19,21^, and insufficient counselling on newborn care^19,21,36^. Women also reported harmful practices, such as the use of external fundal pressure to facilitate birth^18,29^ and the performance of episiotomy, as well as the suturing of episiotomies and vaginal tears, without anaesthesia^18,41^. Although episiotomy was unpopular among women, many accepted the procedure because they believed it was necessary to save their lives^23^. Women experienced pain during labor and the postpartum period regardless of the mode of birth, reflecting challenges related to inadequate pain management^19,20,23,27,31^:

*‘I really tried to lie still when the pains came. They [providers in the labor room] had told me that if I thrashed around too much, I would twist the neck of my baby inside the womb. I really tried to lie as still as possible, but it was so difficult!’* ^18^

### Environment and resource availability

Seven studies contributed to this subcategory^18,21-23,28,30,34^. Opportunities and challenges for a positive childbirth experience were also related to the environment and resource availability within health facilities. Several studies showed that the cleanliness of the health facility was important to women^18,22,23,28,30^. One study from Sri Lanka involving 1265 women reported that 88.8%, 88.0%, and 81.1% of women were highly satisfied with the availability of resources, the space available in the facility, and the cleanliness of the toilets, respectively^30^. Women also associated adequate infrastructure and the availability of basic resources with a positive childbirth experience, including access to beds, clean facilities, and essential amenities within the health facility^18,21-23,28,30,34^. Challenges included limited space in rooms^34^, overcrowding^18^, and unclean environments^22,28^. Studies also showed that women were dissatisfied with facilities lacking access to water^28^, insufficient bed availability^18,23^, the absence of heating facilities during winter^18^, and the lack of a call button to request assistance^21^:

*‘…We did not have any bed, (we stayed) in the corridor itself. So when I was very tired, my mother-in-law spread a shawl on the floor for me to rest for some time … I wish they had given us a bed.* ^18^

### Emotional and physical support

Opportunities and challenges related to the provision of emotional and physical support are described under the subcategories: 1) Strengthening women’s capabilities, 2) Birth companions, and 3) Communication.

### Strengthening women’s capabilities

Fourteen studies contributed to this subcategory^17-23,26,28,29,^ ^31,35,38,40^. Encouragement and supportive care from families, healthcare providers, and fellow women were important opportunities contributing to a positive childbirth experience, as they strengthened women’s ability to cope with labor and birth and to care for their newborns^17-22,40^. Studies revealed that women were satisfied with the supportive care they received^26,29,31,35^. Women also benefited from spiritual support when their religious and cultural needs were met^17,20,38^. During labor, healthcare providers comforted, motivated, and reassured women and helped them maintain their dignity^20,22,23^. Women received emotional and practical support from healthcare providers, fellow women, husbands, relatives, and family members^18-22,40^.

Women reported confidence in healthcare providers^20^, appreciated the psychological support they received^19^, and felt empowered to care for their babies^19^. However, studies also identified challenges related to inattentive healthcare providers who failed to show empathy for women’s labor pain or provide comfort and reassurance^21,23^. A lack of supportive care^9,21,23,40^ reduced women’s trust in healthcare providers^28^. Even when supportive care did not fully meet women’s expectations, women prioritized survival over their childbirth experiences, as the importance of survival overshadowed the challenges they encountered:

*‘Surprisingly, those who reported poor interpersonal care did not consider the overall quality of care to be poor, often because they had been “saved”.’* ^23^

### Birth companions

Eight studies contributed to this subcategory^18,20-22,28,31,33,41^. Birth companions, such as husbands, female relatives, and friends, represented an important opportunity for a positive childbirth experience by providing emotional and physical support. Women were allowed to have a birth companion during all stages of labour^18,20,21,31,33^, only during the first stage of labour^22^, or not at all^41^. A study from Nepal reported that fewer than 20% of 54919 women had a companion of their choice during labor^33^. The presence of a birth companion was perceived by women as contributing to a positive childbirth experience^31^. However, women also faced challenges related to the presence of birth companions during labor, as being seen in an exposed and vulnerable state was perceived as potentially affecting future relationships^18^. Some women asked their companions to leave, even when they were uncomfortable expressing their wishes in their presence^21^. Despite these challenges, women expressed that the presence of a family member in the labor room^28^, preferably their husbands^22^, would enhance their childbirth experience:

*‘When it is very painful, I bit his clothes. I feel so calm when he stands beside me. In every delivery process, he is just enough. He massages and prays for me continuously. And it makes me strong.* ^20^

### Communication

Eighteen studies contributed to this subcategory^18-22,24,26,27,29-31,33,34,36,38,40-42^. Women were satisfied with communication with healthcare providers and the information they received^20,26,27,29,33,34,38^. Opportunities for a positive childbirth experience included positive interactions with healthcare providers, receiving information, and discussing concerns related to the physiological process of childbirth^20^ and medical procedures^29,31,33,36^, as well as communicating about the care of their newborn^22,30,40^. Women’s experiences of communication ranged from effective and supportive communication to a complete absence of communication^18,19,21,24,27,29-31,33,36,38,40,41^, with poor communication representing a significant challenge. Only 14.6% of 405 women in a study from Nepal received information at discharge^27^, and 80.4% of 62926 women in Nepal received inadequate information regarding their care^36^. Furthermore, 85.8% of 400 women in Sri Lanka did not receive information about medications administered during childbirth^31^. Although women received physical care, they were often not given explanations as to why procedures such as cesarean sections were necessary, which led some women to become suspicious of the care provided^21^. Women also encountered language barriers that hindered communication^24,41^. Some studies reported that women were able to discuss their concerns and preferences and that healthcare providers responded to them^29,34,38^, whereas other studies found that women were unable to do so^36,41^. Women attributed poor or inadequate communication to the personalities of individual healthcare providers^21^, as well as to staff workload and overcrowding within the ward^18,19^. Women expressed a desire for positive interactions with healthcare providers and clear explanations regarding medical interventions^20,42^, as these were considered important components of a positive childbirth experience.

### Adherence to principles of women’s rights

Opportunities and challenges related to respect, consent, autonomy, dignity, privacy, and equality of care during childbirth were associated with the principles of women’s rights. These findings are further described under the subcategories: 1) Respectful maternity care, and 2) Equality of care.

### Respectful maternity care

Eighteen studies contributed to this subcategory^17,18,21-24,27-33,36,38,39,41,42^. An opportunity for a positive childbirth experience arose when women experienced privacy^21,23^, dignity^42^, and respectful care^31-33,38^. Women were particularly satisfied when privacy was maintained during physical examinations^30,33,38,29^ and when their consent was sought before procedures were performed^29,31,42^. Lack of privacy and confidentiality^18,21,27,32,39,41^ were reported as challenges to a positive childbirth experience. Lack of privacy was attributed to the absence of curtains separating women in the labor room^18^ and the high number of healthcare providers present during physical examinations^41^. Non-dignified care included not being addressed by name^18^ and exposure of women’s bodies in the labor room^18,39^ to male healthcare providers, medical students, and family members^18,21^, resulting in feelings of shame and, in some cases, trauma^21^. Women also experienced non-consented care^31,32,41^. Consent was not obtained for postpartum contraceptive use^18,21^ or episiotomy^42^. A study from Sri Lanka reported that 55% of 400 women felt that they had not been involved in decisions regarding their care^31^. Women were not involved in decision-making related to the mode of birth^21^ or the choice of contraceptive methods^18,21^. In some cases, women required their husbands to provide consent for a cesarean section^21^. However, studies from India and Nepal reported that only a few women placed a high value on the opportunity to make informed decisions^18,22^. A qualitative study from India^23^ reported that a woman who was dissatisfied due to an episiotomy and experiences of verbal abuse nevertheless described her overall childbirth experience as positive because she had been ‘saved’ by the healthcare provider. Some studies reported that women received respectful and non-abusive care^17,28,29,31-33,36,38^, whereas others documented experiences of physical^17,18,24,31,41,42^, verbal^17,18,24,42^, sexual^24^, and emotional abuse^17,24^ during childbirth:

*‘They use dirty words (about sex leading to pregnancy and childbirth). This is a private matter, and everyone has to sleep with a man to have a baby. However, it should not be spoken like this in front of all to hear* …^18^

### Equality of care

Eleven studies contributed to this subcategory^17,18,23,24,29,32,^ ^36,38,39,41,42^. Opportunities for a positive childbirth experience included non-discriminatory care^23,24^ and the absence of refusal of care^17,29,32,36,38,39,41,42^. Women expressed that receiving non-discriminatory care was important for a positive childbirth experience^42^. One study from Nepal reported that none of the 4296 women in the study population was refused care because of an inability to pay^38^. Another study from Nepal found that only 1.5% of 62926 women were refused care due to an inability to pay^36^. Challenges to a positive childbirth experience included discrimination and the refusal of care through neglect^32^, withholding of information^18^, and either refusing to perform procedures or performing them in a painful manner^18^. In India, 10.2% of 305 women experienced abandonment during childbirth^32^, while 9.7% of 2639 women in another Indian study^29^ and 3.9% of 305 women in Nepal^32^ reported experiencing discrimination by healthcare providers. Women who had personal connections or relatives working within the health facility were sometimes treated preferentially^24^. Challenges included discrimination, neglect, refusal of care, withholding of information, and unequal treatment based on economic status, language, or sociocultural background^17,18,24,29,32,39,41,42^. Nevertheless, many remained grateful to healthcare providers for assisting them through a safe birth^24^, demonstrating how survival was often prioritized over the challenges encountered during childbirth. The importance of survival frequently overshadowed experiences of discrimination and mistreatment:

*‘Additionally, regardless of their negative experiences, the women considered the safe births of their children to be of most importance…* ^24^

## DISCUSSION

In this integrative review of childbirth experiences in health facilities across South-East Asia, the findings revealed that women prioritized the survival of themselves and their newborns over their childbirth experiences, as the importance of survival often overshadowed both the opportunities and challenges encountered during childbirth. Women reported opportunities for positive childbirth experiences through good-quality care provided by competent and attentive healthcare providers, evidence-based practices, adequately resourced health facilities, emotional and physical support from healthcare providers, fellow women, and family members, and respectful care free from discrimination. Challenges included inattentive care, a lack of kangaroo mother care, insufficient education and counselling on breastfeeding and newborn care, inadequate pain management, unclean health facilities, limited access to birth companions, ineffective communication, and various forms of abuse and discrimination. Women often appeared to tolerate poor-quality care and rarely reported mistreatment through formal mechanisms. Nonetheless, they identified several areas where improvements could enhance their childbirth experiences. The following discussion focuses on the key findings of this review.

Our key finding was that women prioritized survival over their childbirth experiences when giving birth in health facilities. This finding may be understood in the context of the WHO-defined South-East Asia Region, where substantial progress has been made in reducing maternal and newborn mortality, but important inequalities in access to high-quality maternity care remain. In settings where safe childbirth cannot always be taken for granted, women may place greater value on survival and positive clinical outcomes than on interpersonal aspects of care. A systematic review found that maternal and newborn survival and health outcomes strongly influenced women’s satisfaction with childbirth care, supporting this finding^43^. The survival of women and newborns was closely linked to the quality of care received during childbirth. Quality maternity care depends on the provision of evidence-based practices and appropriate clinical care, rather than on the professional title of the individual healthcare provider^13^. This is particularly relevant in settings where doctors, nurses, nurse-midwives, and midwives work together in labor and birth units. Beyond the findings of this review, previous research has shown that midwives educated according to international standards have been identified as a safe, effective and cost-efficient workforce. Investments in midwife-led care have the potential to avert substantial proportions of maternal deaths, newborn deaths and stillbirths while improving women’s experiences of care^13,44,45^. Our review identified that care delivered by competent healthcare providers within adequately resourced health facilities contributed to higher levels of satisfaction among women. Similar findings have been reported in a systematic review examining women’s satisfaction with maternity care across diverse settings^43^. Based on our findings and previous research, evidence-based practices that improve health outcomes and save lives appear to be important components of a positive childbirth experience^10,46^. Continued efforts to support implementation of these practices across different maternity care settings may therefore be beneficial. Although several studies reported that healthcare providers valued evidence-based practices, implementation remained inconsistent^47-50^. Furthermore, the WHO recommendations on intrapartum care for a positive childbirth experience have not yet been fully implemented across all settings in the region^10^. Disrespectful care and unequal treatment contributed substantially to negative childbirth experiences in this review. Disrespect and discrimination during childbirth have been widely documented both within South-East Asia^51,52^ and in countries outside the region^53-55^, occurring in both low-resource and high-resource settings^56,57^. Previous research has shown that some women perceive disrespectful maternity care as a normal part of childbirth^58^ and may tolerate abusive treatment when they believe it contributes to a safe birth outcome^59^, consistent with the findings of this review. This highlights the importance of increasing awareness of women’s rights and strengthening respectful maternity care to reduce the normalization and acceptance of mistreatment during childbirth. The presence of a birth companion during labor, childbirth, and the postnatal period may further support women’s rights and contribute to respectful maternity care. Women in this review valued companionship during childbirth, consistent with findings from other studies^60-62^. However, our review showed that overcrowding and limited privacy often restricted the presence of birth companions in health facilities where negative childbirth experiences may contribute to women choosing home birth in subsequent pregnancies despite recommendations for facility-based birth^4^. Overall, the findings suggest that quality of care, emotional and physical support, and respectful maternity care are important aspects of women’s childbirth experiences. The included studies identified several factors that may support positive childbirth experiences, including implementation of evidence-based practices, effective communication, companionship during labor and birth, and respect for women’s rights. These findings are consistent with previous evidence from India, where respectful maternity care, supportive companionship, effective communication, and the removal of financial barriers were identified as important factors influencing positive childbirth experiences^62^. Midwives may play an important role in supporting these aspects of care, particularly for women with uncomplicated pregnancies and births, consistent with WHO recommendations for a positive childbirth experience^10^.

### Limitations

As this study was conducted as an integrative review. The review was not prospectively registered in PROSPERO or another review registry. The main limitation is that title/abstract screening and full-text eligibility assessment were conducted by one reviewer rather than by two independent reviewers. Due to resource constraints, dual-independent screening was not undertaken. Although uncertainties were discussed among the authors, this may have increased the risk of study selection bias. Only studies published in English were included, which may have introduced language bias. The included studies were heterogeneous with regard to study design, setting, populations, and outcome measures, which may have limited comparability across studies. Searches were conducted in two databases and supplemented by reference list screening; therefore, relevant studies indexed in other databases may have been missed. Grey literature was not systematically searched, and publication bias cannot be excluded. Furthermore, four countries within the WHO-defined South-East Asia Region were not represented in the included studies because no eligible published research was identified. Additional research is therefore needed to explore the childbirth experiences of women in these countries and to provide a more comprehensive understanding of the region.

## CONCLUSION

The findings of this integrative review reveal that women prioritize the survival of themselves and their newborns over their childbirth experiences. The findings suggest that positive childbirth experiences may be supported by evidence-based practices, the presence of a birth companion of choice, continuous availability of healthcare providers, environments that facilitate privacy, and respectful maternity care provided by competent healthcare professionals. Future research should explore effective strategies to improve women’s childbirth experiences, including interventions that strengthen respectful maternity care, communication, emotional support, and the implementation of evidence-based practices across diverse maternity care settings.

## Supplementary Material



## Data Availability

The datasets used and analyzed during this review are available from the corresponding author upon reasonable request. Relevant data are included in this article and the Supplementary file.
