# Supplementary material for: From survival to positive childbirth experiences: An integrative review of facility-based maternity care in South-East Asia
Source: Eur J Midwifery. 2026 Oct 22;10:10.18332/ejm/240344. doi: 10.18332/ejm/240344 (PMC13690049; doi:10.18332/ejm/240344)
Supplement: Supplementary file 1 [file EJM-10-49-s1.pdf]

EUROPEAN JOURNAL
OF MIDWIFERY

Supplementary file

© 2026 Arulpragassam E. D. et al.

DOI:
10.18332/ejm/240344

The content has been provided by the author(s) and has not been reviewed, verified, or endorsed by European Publishing. It may not have undergone peer review. The views, opinions, and recommendations expressed are solely those of the author(s) and do not necessarily reflect the position of European Publishing. European Publishing accepts no responsibility or liability for any consequences arising from the use of, or reliance on, this content.

FULL SEARCH STRATEGY

CINAHL 2022.01.25

	Search Details	Results
#7	#1 AND #2 AND #3 AND #6	652
#6	#4 OR #5	49574
#5	South Asia OR South East Asian	2380
#4	Bangladesh OR Bhutan OR Korea OR India OR Indonesia OR Maldives OR Myanmar OR Sri Lanka OR Thailand OR Timor-Leste OR Nepal OR South East Asia	48517
#3	parturition OR childbirth OR deliveries OR obstetric delivery OR postpartum OR postnatal OR intrapartum	81800
#2	midwife OR midwifery OR midwifery care OR delivery care OR hospital care OR postpartum care OR postnatal unit OR intrapartum care	138562
#1	experiences OR perception OR satisfaction OR attitudes	337831

PubMed 2022.12.12

	Search Details	Results
#5	#1 AND #2 AND #3 AND #4	2212
#4	("midwife s"[All Fields] OR "midwifery"[MeSH Terms] OR "midwifery"[All Fields] OR "midwife"[All Fields] OR "midwifes"[All Fields] OR "midwifing"[All Fields] OR ("midwifery"[MeSH Terms] OR "midwifery"[All Fields]) OR (("midwifery"[MeSH Terms] OR "midwifery"[All Fields]) AND "care"[All Fields]) OR (("deliveries"[All Fields] OR "delivery, obstetric"[MeSH Terms] OR ("delivery"[All Fields] AND "obstetric"[All Fields]) OR "obstetric delivery"[All Fields] OR "delivery"[All Fields]) AND "care"[All Fields]) OR (("hospital s"[All Fields] OR "hospitalisation"[All Fields] OR "hospitalization"[MeSH Terms] OR "hospitalization"[All Fields] OR "hospitalised"[All Fields] OR "hospitalising"[All Fields] OR "hospitality"[All Fields] OR "hospitalisations"[All Fields] OR "hospitalizations"[All Fields] OR "hospitalize"[All Fields] OR "hospitalized"[All Fields] OR "hospitalizing"[All Fields] OR "hospitals"[MeSH Terms] OR "hospitals"[All Fields] OR "hospital"[All Fields]) AND "care"[All Fields]) OR ("postnatal care"[MeSH Terms] OR ("postnatal"[All Fields] AND "care"[All Fields]) OR "postnatal care"[All Fields] OR ("postpartum"[All Fields] AND "care"[All Fields]) OR "postpartum care"[All Fields]) OR (("postnatal"[All Fields] OR "postnatally"[All Fields]) AND "unit"[All Fields]) OR ("intrapartum"[All Fields] AND "care"[All Fields])) AND ((y_10[Filter]) AND (humans[Filter]) AND (English[Filter]))	685386

#3	("parturition"[MeSH Terms] OR "parturition"[All Fields] OR "delivery, obstetric"[MeSH Terms] OR ("delivery"[All Fields] AND "obstetric"[All Fields]) OR "obstetric delivery"[All Fields] OR "parturitions"[All Fields] OR "parturitional"[All Fields] OR ("parturition"[MeSH Terms] OR "parturition"[All Fields] OR "childbirth"[All Fields] OR "childbirths"[All Fields]) OR ("deliveries"[All Fields] OR "delivery, obstetric"[MeSH Terms] OR ("delivery"[All Fields] AND "obstetric"[All Fields]) OR "obstetric delivery"[All Fields] OR "delivery"[All Fields]) OR ("delivery, obstetric"[MeSH Terms] OR ("delivery"[All Fields] AND "obstetric"[All Fields]) OR "obstetric delivery"[All Fields] OR ("obstetric"[All Fields] AND "deliveries"[All Fields]) OR "obstetric deliveries"[All Fields]) OR ("postpartum period"[MeSH Terms] OR ("postpartum"[All Fields] AND "period"[All Fields]) OR "postpartum period"[All Fields] OR "postpartum"[All Fields]) OR ("postnatal"[All Fields] OR "postnatally"[All Fields]) OR "intrapartum"[All Fields]) AND ((y_10[Filter]) AND (humans[Filter]) AND (english[Filter]))	308538
#2	("experience"[All Fields] OR "experience s"[All Fields] OR "experiences"[All Fields] OR ("percept"[All Fields] OR "perceptibility"[All Fields] OR "perceptible"[All Fields] OR "perception"[MeSH Terms] OR "perception"[All Fields] OR "perceptions"[All Fields] OR "perceptional"[All Fields] OR "perceptive"[All Fields] OR "perceptiveness"[All Fields] OR "percepts"[All Fields]) OR ("attitude"[MeSH Terms] OR "attitude"[All Fields] OR "attitudes"[All Fields] OR "attitude s"[All Fields]) OR ("personal satisfaction"[MeSH Terms] OR ("personal"[All Fields] AND "satisfaction"[All Fields]) OR "personal satisfaction"[All Fields] OR "satisfaction"[All Fields] OR "satisfactions"[All Fields] OR "satisfaction s"[All Fields])) AND ((y_10[Filter]) AND (humans[Filter]) AND (english[Filter]))	756337
#1	("Bangladesh"[MeSH Terms] OR "Bangladesh"[All Fields] OR "Bangladesh s"[All Fields] OR ("Bhutan"[MeSH Terms] OR "Bhutan"[All Fields] OR "Bhutan s"[All Fields]) OR ("Korea"[MeSH Terms] OR "Korea"[All Fields] OR "Korea s"[All Fields] OR "Koreas"[All Fields]) OR ("India"[MeSH Terms] OR "India"[All Fields] OR "India s"[All Fields] OR "Indias"[All Fields]) OR ("Indonesia"[MeSH Terms] OR "Indonesia"[All Fields] OR "Indonesia s"[All Fields] OR "Indonesias"[All Fields]) OR ("Maldive"[All Fields] OR "Maldives"[All Fields]) OR ("Myanmar"[MeSH Terms] OR "Myanmar"[All Fields] OR "Myanmar s"[All Fields] OR "Myanmars"[All Fields]) OR ("Sri Lanka"[MeSH Terms] OR ("Sri"[All Fields] AND "Lanka"[All Fields]) OR "Sri	452391

	Lanka"[All Fields]) OR ("Thailand"[MeSH Terms] OR "Thailand"[All Fields] OR "Thailand s"[All Fields]) OR ("Timor Leste"[MeSH Terms] OR "timor leste"[All Fields] OR ("Timor"[All Fields] AND "Leste"[All Fields]) OR "Timor Leste"[All Fields]) OR ("Nepal"[MeSH Terms] OR "Nepal"[All Fields] OR "Nepal s"[All Fields]) OR ("asia, Southeastern"[MeSH Terms] OR ("Asia"[All Fields] AND "Southeastern"[All Fields]) OR "Southeastern Asia"[All Fields] OR ("South"[All Fields] AND "East"[All Fields] AND "Asia"[All Fields]) OR "South East Asia"[All Fields]) OR (East, South[Investigator] AND ("Asian"[MeSH Terms] OR "Asian"[All Fields] OR "Asian people"[MeSH Terms] OR ("Asian"[All Fields] AND "people"[All Fields]) OR "Asian people"[All Fields] OR "Asians"[All Fields])) OR ("South"[All Fields] AND ("Asian"[MeSH Terms] OR "Asian"[All Fields] OR "Asian people"[MeSH Terms] OR ("Asian"[All Fields] AND "people"[All Fields]) OR "Asian people"[All Fields] OR "Asians"[All Fields]))) AND ((y_10[Filter]) AND (humans[Filter]) AND (English[Filter]))	

Excluded articles with reasons

Article	Reason for exclusion
Ashish KC, Peterson SS, Gurung R, Skalkidou A, Gautam J, Malla H, Paudel P, Bhattarai K, Joshi N, Tinkari BS, Adhikari S, Shrestha D, Ghimire B, Sharma S, Khanal L, Shrestha S, Graham WJ, Kinney M. The perfect storm: Disruptions to institutional delivery care arising from the COVID-19 pandemic in Nepal. J Glob Health. 2021 May 11;11:05010. doi: 10.7189/jogh.11.05010. PMID: 34055329; PMCID: PMC8141327.	Childbirth experience during COVID
Sahoo KC, Doley C, Negi S, Das S, Verma P, Kanungo S, Pati S. Experiences of Urban Slum-Dwelling Women With Maternal and Child Health Services During COVID-19 Pandemic: A Multi-City Qualitative Study From India. Int J Public Health. 2022 Sep 20;67:1604348. doi: 10.3389/ijph.2022.1604348. PMID: 36204007; PMCID: PMC9530035.	Childbirth experience during COVID
Milkowska-Shibata MA, Aye TT, Yi SM, Oo KT, Khaing K, Than M, Win T, Myo SY, Toe SY, West HS, Ringstad KM, Galarza L, Meng C, Shibata T. Understanding Barriers and Facilitators of Maternal Health Care Utilization in Central Myanmar. Int J Environ Res Public Health. 2020 Feb 25;17(5):1464. doi:10.3390/ijerph17051464. PMID: 32106467; PMCID: PMC7084571.	Home birth
Morrison J, Basnet M, Budhathoki B, Adhikari D, Tumbahangphe K, Manandhar D, Costello A, Groce N. Disabled women's maternal and newborn health care in rural Nepal: a qualitative study. Midwifery. 2014 Nov;30(11):1132-9. doi: 10.1016/j.midw.2014.03.012. Epub 2014 Mar 28. PMID: 24768318; PMCID: PMC4217148	Home birth
Tanaka, K., Kurniasari, N.M.D., Widyanthini, D.N. *et al.* Perception of childbirth experiences of Japanese women in Bali, Indonesia: a qualitative study. *BMC Pregnancy Childbirth* 20, 760 (2020). https://doi.org/10.1186/s12884-020-03466-x	Migrant women
Chu MS, Park M, Kim JA. First childbirth experience of international marriage migrant women in South Korea. Women and Birth. 2017 Aug;30(4):e198–206.	Migrant women
Montagu D, Giessler K, Nakphong MK, Roy KP, Sahu AB, Sharma K, Green C, Sudhinaraset M. Results of a person-centered maternal health quality improvement intervention in Uttar Pradesh, India. PLoS One. 2020 Dec 11;15(12):e0242909. doi: 10.1371/journal.pone.0242909. PMID: 33306689; PMCID: PMC7732121.	Mixed results
Bhattacharya S, Sundari Ravindran TK. Silent voices: institutional disrespect and abuse during delivery among women of Varanasi district, northern India. BMC Pregnancy Childbirth. 2018 Aug 20;18(1):338. doi: 10.1186/s12884-018-1970-3. PMID: 30126357; PMCID: PMC6102865	Mixed results
Akhter S, Dasvarma GL, Saikia U. Reluctance of women of lower socio-economic status to use maternal healthcare services - Does only cost matter? PLoS One. 2020 Sep 29;15(9):e0239597. doi: 10.1371/journal.pone.0239597. PMID: 32991622; PMCID: PMC7523962	Mixed results

Maru S, Bangura AH, Mehta P, Bista D, Borgatta L, Pande S, Citrin D, Khanal S, Banstola A, Maru D. Impact of the roll out of comprehensive emergency obstetric care on institutional birth rate in rural Nepal. BMC Pregnancy Childbirth. 2017 Mar 4;17(1):77. doi: 10.1186/s12884-017-1267-y. PMID: 28259150; PMCID: PMC5336683.	Mixed results
Bhattarai P. Factors Associated with Use of Maternal Health Services in Nepal: Analysis of the 2016 Nepal Demographic and Health Survey. J Nepal Health Res Counc. 2019 Nov 13;17(3):301-307. doi: 10.33314/jnhrc.v17i3.1525. PMID: 31735922.	Mixed results
Singh P, Rasania SK. Determinants of Health-Related Quality of Life among Post-Partum Women Residing in a Resettlement Colony of East Delhi. Indian J Community Health [Internet]. 2022 Jun. 30 [cited 2023 Aug. 8];34(2):188-95. Available from: https://www.iapsmupuk.org/journal/index.php/IJCH/article/view/2306	Mixed results
Phanwichatkul T, Schmied V, Liamputtong P, Burns E. The perceptions and practices of Thai health professionals providing maternity care for migrant Burmese women: An ethnographic study. *Women Birth* . 2022;35(4):e356-e368. doi:10.1016/j.wombi.2021.07.003	Mixed results
C. Corbett, L.C. Callister Giving Birth: the Voices of Women in Tamil Nadu, India The American Journal of Maternal Child Nursing, 37 (2012), pp. 298-305	Mixed results
Chattopadhyay S, Mishra A, Jacob S. "Safe", yet violent? Women's experiences with obstetric violence during hospital births in rural Northeast India. Culture, Health & Sexuality. 2017 Nov 3;20(7):815–29.	Mixed results
Bhattacharyya S, Issac A, Rajbangshi P, Srivastava A, Avan BI. "Neither we are satisfied nor they"-users and provider's perspective: a qualitative study of maternity care in secondary level public health facilities, Uttar Pradesh, India. BMC Health Services Research. 2015 Jun;15(1).	Mixed results
Dey A, Shakya HB, Chandurkar D, Kumar S, Das AK, Anthony J, et al. Discordance in self-report and observation data on mistreatment of women by providers during childbirth in Uttar Pradesh, India. Reproductive Health. 2017 Nov 15;14(1).	Mixed results
Chalise B, Chalise M, Bista B, Pandey AR, Thapa S. Correlates of continuum of maternal health services among Nepalese women: Evidence from Nepal Multiple Indicator Cluster Survey. PLoS One. 2019 Apr 19;14(4):e0215613. doi: 10.1371/journal.pone.0215613. PMID: 31002686; PMCID: PMC6474612.	Not according to aim
Diamond-Smith N, Treleaven E, Murthy N, Sudhinaraset M. Women's empowerment and experiences of mistreatment during childbirth in facilities in Lucknow, India: results from a cross-sectional study. BMC Pregnancy Childbirth. 2017 Nov 8;17(Suppl 2):335. doi: 10.1186/s12884-017-1501-7. PMID: 29143668; PMCID: PMC5688442	Not according to aim
Kim JE, Na BJ, Kim HJ, Lee JY. Why Women Living in an Obstetric Care Underserved Area Do Not Utilize Their Local Hospital Supported by Korean Government for Childbirth. Asian Nurs Res (Korean Soc Nurs Sci). 2016 Sep;10(3):221-227. doi: 10.1016/j.anr.2016.07.001. Epub 2016 Jul 17. PMID: 27692252	Not according to aim

Devkota HR, Clarke A, Murray E, Groce N. Do experiences and perceptions about quality of care differ among social groups in Nepal? : A study of maternal healthcare experiences of omen with and without disabilities, and Dalit and non-Dalit women. PLoS One. 2017 Dec 19;12(12):e0188554. doi:10.1371/journal.pone.0188554. PMID: 29261691; PMCID: PMC5736179.	Not according to aim
Pervin J, Venkateswaran M, Nu UT, Rahman M, O'Donnell BF, Friberg IK, Rahman A, Frøen JF. Determinants of utilization of antenatal and delivery care at the community level in rural Bangladesh. PLoS One. 2021 Sep 28;16(9):e0257782. doi:10.1371/journal.pone.0257782. PMID: 34582490; PMCID: PMC8478219.	Not according to aim
Krishnamoorthy Y, Majella MG. Determinants of postnatal care coverage among mothers and new-borns in India: Evidence from a nationally representative survey. Int J Health Plann Manage. 2021 Jul;36(4):1276-1286. Doi: 10.1002/hpm.3179. Epub 2021 Apr 17. PMID: 33866592.	Not according to aim
Lee DE, Suh HW, Park HS, Youn I, Park M, Seo J. Mothers' Experiences of Childbirth and Perspectives on Korean Medicine-Based Postpartum Care in Korea: A Qualitative Study. Int J Environ Res Public Health. 2022 Apr 27;19(9):5332. doi: 10.3390/ijerph19095332. PMID: 35564730; PMCID: PMC9105879	Not according to aim
Saxena M, Srivastava A, Dwivedi P, Bhattacharyya S. Is quality of care during childbirth consistent from admission to discharge? A qualitative study of delivery care in Uttar Pradesh, India. PLoS One. 2018 Sep 27;13(9):e0204607. doi: 10.1371/journal.pone.0204607. PMID: 30261044; PMCID: PMC6160099	Not according to aim
Kesuma ZM, Chongsuvivatwong V. Comparison of satisfaction with maternal health-care services using different health insurance schemes in aceh province, Indonesia. Indian J Public Health. 2016 Jul Sep;60(3):195-202. doi: 10.4103/0019-557X.189013. PMID: 27561398.	Not according to aim
Choulagai BP, Onta S, Subedi N, Bhatta DN, Shrestha B, Petzold M, Krettek A. A cluster-randomized evaluation of an intervention to increase skilled birth attendant utilization in mid- and far-western Nepal. Health Policy Plan. 2017 Oct 1;32(8):1092-1101. doi: 10.1093/heapol/czx045. PMID: 28475754; PMCID: PMC5886087.	Not according to aim
Tschirhart N, Jiraporncharoen W, Angkurawaranon C, Hashmi A, Nosten S, McGready R, Ottersen T. Choosing where to give birth: Factors influencing migrant women's decision making in two regions of Thailand. PLoS One. 2020 Apr 2;15(4):e0230407. doi: 10.1371/journal.pone.0230407. PMID: 32240176; PMCID: PMC7117675	Not according to aim
Mondal D, Karmakar S, Banerjee A. Women's autonomy and utilization of maternal healthcare in India: Evidence from a recent national survey. PLoS One. 2020 Dec 9;15(12):e0243553. doi: 10.1371/journal.pone.0243553. PMID: 33296428; PMCID: PMC7725295.	Not according to aim
Huq NL, Ahmed A, Haque NA, Hossaine M, Uddin J, Ahmed F, Quaiyum MA. Effect of an integrated maternal health intervention on skilled provider's care for maternal health in remote rural areas of Bangladesh: a pre and post study. BMC Pregnancy Childbirth. 2015 Apr 28;15:104. doi: 10.1186/s12884-015-0520-5. PMID: 25928009; PMCID: PMC4429323.	Not according to aim

Mahala U, Mehta S. Maternal health outcomes following the implementation of Janani Shishu Suraksha Yojana (JSSY) at a tertiary healthcare center in Rajasthan. Int J Gynaecol Obstet. 2017 Dec;139(3):297-300. doi: 10.1002/ijgo.12316. Epub 2017 Sep 28. PMID: 28865163	Not according to aim
Taleb F, Perkins J, Ali NA, Capello C, Ali M, Santarelli C, Hoque DM. Transforming maternal and newborn health social norms and practices to increase utilization of health services in rural Bangladesh: a qualitative review. BMC Pregnancy Childbirth. 2015 Mar 29;15:75. doi: 10.1186/s12884-015-0501-8. PMID: 25886165; PMCID: PMC4391089.	Not according to aim
Kovavisarach E, Sukontaman W. Preferred Route of Delivery of Thai Pregnant Women. J Med Assoc Thai. 2017 Feb;100 Suppl 1:S131-5. PMID: 29927231.	Not according to aim
Sapkota S, Kobayashi T, Takase M. Impact on perceived postnatal support, maternal anxiety and symptoms of depression in new mothers in Nepal when their husbands provide continuous support during labour. Midwifery. 2013 Nov;29(11):1264-71. doi: 10.1016/j.midw.2012.11.010. Epub 2013 Feb 15. PMID: 23415367	Not according to aim
Paudel G, Yadav UN, Thakuri SJ, Singh JP, Marahatta SB. Utilization of services for institutional deliveries in Gorkha District. J Nepal Health Res Counc. 2016 Sep;14(34):202-206. PMID: 28327687.	Not according to aim
Suwanrath C, Chunuan S, Matemanosak P, Pinjaroen S. Why do pregnant women prefer cesarean birth? A qualitative study in a tertiary care center in Southern Thailand. BMC Pregnancy Childbirth. 2021 Jan 6;21(1):23. doi: 10.1186/s12884-020-03525-3. PMID: 33407245; PMCID: PMC7789505	Not according to aim
Khanam R, Creanga AA, Koffi AK, Mitra DK, Mahmud A, Begum N, Moin SM, Ram M, Quaiyum MA, Ahmed S, Saha SK, Baqui AH; Projahnmo Study Group in Bangladesh. Patterns and Determinants of Care-Seeking for Antepartum and Intrapartum Complications in Rural Bangladesh: Results from a Cohort Study. PLoS One. 2016 Dec 20;11(12):e0167814. doi: 10.1371/journal.pone.0167814. PMID: 27997537; PMCID: PMC5172566	Not according to aim
Toe SY, Higuchi M, Htay SS, Hamajima N. Health care seeking behaviors regarding maternal care and the associated factors among married women in Naung Cho Township, Myanmar. Nagoya J Med Sci. 2021 Nov;83(4):727-740. doi: 10.18999/nagjms.83.4.727. PMID: 34916717; PMCID: PMC8648524.	Not according to aim
Andriani H, Rahmawati ND, Fauzia S, Kosasih RI. Population-Based Study on the Maternal-Newborn-Child Health Continuum of Care: Evidence From Lower-Middle-Income Countries in Southeast Asia. Asia Pac J Public Health. 2022 Jul;34(5):547-556. doi: 10.1177/10105395221088615. Epub 2022 Apr 8. PMID: 35392673.	Not according to aim
Karkee R, Lee AH, Pokharel PK. Women's perception of quality of maternity services: a longitudinal survey in Nepal. BMC Pregnancy Childbirth. 2014 Jan 24;14:45. doi: 10.1186/1471-2393-14-45. PMID: 24456544; PMCID: PMC3902186	Not according to aim
Kothavale A, Meher T. Level of completion along continuum of care for maternal, newborn and child health services and factors associated with it among women in India: a population-based cross-sectional study. BMC Pregnancy Childbirth. 2021 Oct 27;21(1):731. doi: 10.1186/s12884-021-04198-2. PMID: 34706680; PMCID: PMC8554854.	Not according to aim

Aryastami NK, Mubasyiroh R. Traditional practices influencing the use of maternal health care services in Indonesia. PLoS One. 2021 Sep 10;16(9):e0257032. doi: 10.1371/journal.pone.0257032. PMID: 34506525; PMCID: PMC8432883.	Not according to aim
Bruce SG, Blanchard AK, Gurav K, Roy A, Jayanna K, Mohan HL, Ramesh BM, Blanchard JF, Moses S, Avery L. Preferences for infant delivery site among pregnant women and new mothers in Northern Karnataka, India. BMC Pregnancy Childbirth. 2015 Feb 27;15:49. doi: 10.1186/s12884-015-0481-8. PMID: 25884166; PMCID: PMC4345019.	Not according to aim
Rao P, R R, Bethou A, Bhat V, C P. Does Kangaroo Mother Care Reduce Anxiety in Postnatal Mothers of Preterm Babies? - A Descriptive Study from a Tertiary Care Centre in South India. J Nepal Health Res Counc. 2019 Apr 28;17(1):42-45. doi: 10.33314/jnhrc.1228. PMID: 31110375.	Not according to aim
Lodha P, Jahangir T, Karia S, DeSousa A, Appasani R, Withers M. Perceptions of perinatal depression among low-income mothers and families in Mumbai, India. Asian J Psychiatr. 2022 May;71:103048. doi: 10.1016/j.ajp.2022.103048. Epub 2022 Feb 23. PMID: 35272254.	Not according to aim
Rammohan A, Goli S, Saroj SK, Jaleel CPA. Does engagement with frontline health workers improve maternal and child healthcare utilisation and outcomes in India? Hum Resour Health. 2021 Apr 1;19(1):45. doi: 10.1186/s12960-021-00592-1. PMID: 33794920; PMCID: PMC8017836.	Not according to aim
Rupani MP, Patel PM, Meena PR, Patel PP, Patel PA, Paragda PK. Regular Antenatal Care Visits Predict Good Knowledge Among Post-natal Mothers Regarding Entitlements of Health Programs in Western India. Int J Health Policy Manag. 2019 Aug 1;8(8):467-473. doi: 10.15171/ijhpm.2019.28. PMID: 31441286; PMCID: PMC6706960.	Not according to aim
Anik AI, Ghose B, Rahman MM. Relationship between maternal healthcare utilisation and empowerment among women in Bangladesh: evidence from a nationally representative cross-sectional study. BMJ Open. 2021 Aug 13;11(8):e049167. doi: 10.1136/bmjopen-2021-049167. PMID: 34389576; PMCID: PMC8365820.	Not according to aim
Haider MR, Qureshi ZP, Khan MM. Effects of women's autonomy on maternal healthcare utilization in Bangladesh: Evidence from a national survey. Sex Reprod Healthc. 2017 Dec;14:40-47. doi: 10.1016/j.srhc.2017.09.002. Epub 2017 Sep 18. PMID: 29195633.	Not according to aim
Jayanthi TP, Suresh S, Padmanaban P. Primary health centres: preferred option for birthing care in tamilnadu, India, from users' perspectives. J Health Popul Nutr. 2015 Mar;33(1):177-86. PMID: 25995734; PMCID: PMC4438661.	Not according to aim
Gai Tobe R, Islam MT, Yoshimura Y, Hossain J. Strengthening the community support group to improve maternal and neonatal health seeking behaviors: A cluster-randomized controlled trial in Satkhira District, Bangladesh. PLoS One. 2019 Feb 28;14(2):e0212847. doi: 10.1371/journal.pone.0212847. PMID: 30817784; PMCID: PMC6394907.	Not according to aim
Ferdos J, Rahman MM. Maternal experience of intimate partner violence and low birth weight of children: A hospital-based study in Bangladesh. PLoS One. 2017 Oct 26;12(10):e0187138. doi: 10.1371/journal.pone.0187138. PMID: 29073222; PMCID: PMC5658163.	Not according to aim

Rai RK, Singh PK, Kumar C. Is the use of maternal healthcare among prospective mothers higher in households that have experienced maternal death? Evidence from India. Health Policy Plan. 2016 Sep;31(7):844-52. doi: 10.1093/heapol/czv140. Epub 2016 Feb 9. PMID: 26864163.	Not according to aim
Smittenaar P, Ramesh BM, Jain M, Blanchard J, Kemp H, Engl E, Isac S, Anthony J, Prakash R, Gothalwal V, Namasivayam V, Kumar P, Sgaier SK. Bringing Greater Precision to Interactions Between Community Health Workers and Households to Improve Maternal and Newborn Health Outcomes in India. Glob Health Sci Pract. 2020 Sep 30;8(3):358-371. doi: 10.9745/GHSP-D-20-00027. PMID: 33008853; PMCID: PMC7541124.	Not according to aim
Shahabuddin A, Nöstlinger C, Delvaux T, Sarker M, Delamou A, Bardají A, Broerse JE, De Brouwere V. Exploring Maternal Health Care-Seeking Behavior of Married Adolescent Girls in Bangladesh: A Social-Ecological Approach. PLoS One. 2017 Jan 17;12(1):e0169109. doi: 10.1371/journal.pone.0169109. PMID: 28095432; PMCID: PMC5240914.	Not according to aim
Yaya S, Bishwajit G, Ekholuenetale M. Factors associated with the utilization of institutional delivery services in Bangladesh. PLoS One. 2017 Feb 13;12(2):e0171573. doi: 10.1371/journal.pone.0171573. PMID: 28192478; PMCID: PMC5305190.	Not according to aim
Sharma G, Penn-Kekana L, Halder K, Filippi V. An investigation into mistreatment of women during labour and childbirth in maternity care facilities in Uttar Pradesh, India: a mixed methods study. Reprod Health. 2019 Jan 23;16(1):7. doi: 10.1186/s12978-019-0668-y. PMID: 30674323; PMCID: PMC6345007.	Not according to aim
Singh PK, Kumar C, Rai RK, Singh L. Factors associated with maternal healthcare services utilization in nine high focus states in India: a multilevel analysis based on 14 385 communities in 292 districts. Health Policy Plan. 2014 Aug;29(5):542-59. doi: 10.1093/heapol/czt039. Epub 2013 Jun 18. PMID: 23783832.	Not according to aim
Dhar D, McDougal L, Hay K, Atmavilas Y, Silverman J, Triplett D, Raj A. Associations between intimate partner violence and reproductive and maternal health outcomes in Bihar, India: a cross-sectional study. Reprod Health. 2018 Jun 19;15(1):109. doi: 10.1186/s12978-018-0551-2. PMID: 29921276; PMCID: PMC6009820.	Not according to aim
Brar AS, Hedt-Gauthier BL, Hirschhorn LR. Mixed Methods Lot Quality Assurance Sampling: A novel, rapid methodology to inform equity focused maternal health programming in rural Rajasthan, India. PLoS One. 2021 Apr 29;16(4):e0250154. doi: 10.1371/journal.pone.0250154. PMID: 33914763; PMCID: PMC8084134.	Not according to aim
Shukla A, Acharya R, Kumar A, Mozumdar A, Aruldas K, Saggurti N. Client-provider interaction: understanding client experience with family planning service providers through the mystery client approach in India. Sex Reprod Health Matters. 2020 Dec;28(1):1822492. doi: 10.1080/26410397.2020.1822492. PMID: 33054696; PMCID: PMC7566859.	Not according to aim

Kim AR, Tak YR, Shin YS, Yun EH, Park HK, Lee HJ. Mothers' Perceptions of Quality of Family-Centered Care and Environmental Stressors in Neonatal Intensive Care Units: Predictors of and Relationships with Psycho-emotional Outcomes and Postpartum Attachment. *Matern Child Health J* . 2020;24(5):601-611. doi:10.1007/s10995-020-02876-9	Not according to aim
Khonsung P, Yimyam S, Xuto P, Chaloumsuk N. Factors Predicting Exclusive Breastfeeding among Thai Adolescent Mothers at 6-months Postpartum. PRIJNR [Internet]. 2020 Dec. 7 [cited 2023 Aug. 9];25(1):34-47. Available from: https://he02.tci-thaijo.org/index.php/PRIJNR/article/view/239919	Not according to aim
Yoo H, Ahn S, Park S, Kim J, Oh J, Koh M. Factors influencing prenatal and postpartum depression in Korea: a prospective cohort study. *Korean J Women Health Nurs* . 2021;27(4):326-336. doi:10.4069/kjwhn.2021.11.17	Not according to aim
Mayra K, Matthews Z, Padmadas SS. Why do some health care providers disrespect and abuse women during childbirth in India?. *Women Birth* . 2022;35(1):e49-e59. doi:10.1016/j.wombi.2021.02.003	Not according to aim
Myo T, Hong SA, Thepthien B, Hongkrailert N. Prevalence and Factors Associated with Postpartum Depression in Primary Healthcare Centres in Yangon, Myanmar. *The Malaysian journal of medical sciences* . 2021;28:71-86.	Not according to aim
Baria H, Amipara T, Nayak S. A study on postpartum depression and its association with infant feeding practices and infant nutritional status among mothers attending the anganwadi centers of Valsad district, Gujarat, India. Indian Journal of Community Medicine. 2020;45(3):299.	Not according to aim
Sindhuri R, Nancy S, Arunagirinathan A, Dongre A. Breastfeeding positioning and attachment among postnatal mothers: A mixed methods study in a tertiary care hospital in Puducherry, South India. Indian Journal of Community Medicine. 2022;47(1):120.	Not according to aim
Akhter S, Schech S. Choosing caesareans? The perceptions and experiences of childbirth among mothers from higher socio-economic households in Dhaka. Health Care for Women International. 2018 Sep 24;39(11):1177–92.	Not according to aim
Andrea Katryn Blanchard, Bruce S, Krishnamurthy Jayanna, Kaveri Gurav, Mohan HL, Avery L, et al. An Exploration of Decision-Making Processes on Infant Delivery Site from the Perspective of Pregnant Women, New Mothers, and Their Families in Northern Karnataka, India. Maternal and Child Health Journal. 2015 Feb 5;19(9):2074–80.	Not according to aim
Liblub S, Gum L, Bazargan M. How do pregnant women perceive the role of the midwife in Thailand? A descriptive study. Journal of Asian Midwives (JAM) [Internet]. 2020 Dec 1 [cited 2023 Aug 19];7(2):33–47. Available from: https://ecommons.aku.edu/jam/vol7/iss2/4/	Not according to aim
Pardosi JF, Parr N, Muhidin S. INEQUITY ISSUES AND MOTHERS' PREGNANCY, DELIVERY AND EARLY-AGE SURVIVAL EXPERIENCES IN ENDE DISTRICT, INDONESIA. Journal of Biosocial Science [Internet]. 2014 Dec 15; Available from: https://doi.org/10.1017/s0021932014000522	Not according to aim
Yuenyong S, O'Brien B, Jirapeet V. Effects of Labor Support from Close Female Relative on Labor and Maternal Satisfaction in a Thai Setting. Journal of Obstetric, Gynecologic & Neonatal Nursing. 2012 Jan;41(1):45–56.	Not according to aim

Contractor SQ, Das A, Dasgupta J, Van Belle S. Beyond the template: the needs of tribal women and their experiences with maternity services in Odisha, India. International Journal for Equity in Health. 2018 Sep 24;17(1).	Not according to aim
Ansari H, Yeravdekar R. Respectful maternity care: A national landscape review. Natl Med J India. 2019 Sep-Oct;32(5):290-293. doi:10.4103/0970-258X.295957. PMID: 32985445.	Review article
Shrivastava S, Sivakami M. Evidence of “obstetric violence” in India: an integrative review. Journal of Biosocial Science. 2019 Nov 14;52(4):610–28.	Review article
Rajbanshi S, Norhayati MN, Nik Hazlina NH. Perceptions of Good-Quality Antenatal Care and Birthing Services among Postpartum Women in Nepal. Int J Environ Res Public Health. 2021 Jun 26;18(13):6876. doi: 10.3390/ijerph18136876. PMID: 34206868; PMCID: PMC8297004.	Unknown place of birth
King R, Jones L. Timorese women’s experiences and expectations of skilled birth attendance in Timor-Leste: A qualitative inquiry. Sexual & Reproductive Healthcare. 2022 Sep;33:100761.	Unknown place of birth
